# To What Extent Does Medical Training Alter Students’ Ideas Concerning Suicide?

**DOI:** 10.1007/s40596-026-02304-0

**Published:** 2026-02-07

**Authors:** Servet Aker, Mustafa Kürşat Şahin, Özlem Mıdık

**Affiliations:** Ondokuz Mayıs University Faculty of Medicine, Samsun, Turkey

**Keywords:** Suicide, Stigmatization, Medical education

## Abstract

**Objective:**

Given the critical role of medical students in future healthcare settings, understanding their attitudes towards suicide is important. The purpose of this research was to determine medical faculty students’ levels of knowledge concerning suicide and their attitudes toward the stigmatization of suicide, and to evaluate the effect of medical training on those attitudes.

**Methods:**

This cross-sectional study was performed with 684 students at the Ondokuz Mayıs University Medical Faculty between March and May 2024. Data were collected using an online questionnaire created via Google Forms. The form includes the Literacy of Suicide Scale (LOSS) and the Stigma of Suicide Scale (SOSS).

**Results:**

The students’ mean LOSS score was 17.17 ± 0.13, and their mean SOSS Stigma sub-dimension score was 67.95 ± 0.79. The findings indicated that medical students have a higher knowledge level regarding suicide than the general Turkish population. The knowledge levels of medical students increase significantly from the first year to the fourth year, coinciding with their progression to clinical training. Alongside this increase, there is a significant decrease in stigmatizing attitudes toward suicide among fourth-year students compared to first-year students. However, no significant further improvement in knowledge or reduction in stigma was observed once students enter clinical training in their sixth year and begin interacting with or treating patients who have attempted suicide, compared to the fourth year.

**Conclusions:**

Crucially, the study demonstrated that the transition to hands-on clinical practice, including psychiatry internships and direct contact with suicidal patients, did not yield the expected advancements in suicide literacy or attitudes, highlighting a significant deficiency in the current clinical training model.

While suicide is a pressing global health issue, the attitudes of future physicians shaped by their training remain a critical yet underexplored factor in prevention efforts, particularly in culturally specific contexts like Türkiye. It is the fourth leading cause of death among individuals aged 15–29, with over 700,000 deaths annually [1]. In Türkiye, 2022 witnessed 4,146 suicides, resulting in a crude suicide rate of 4.88 per 100,000 [2]. Despite its prevalence, suicide remains a deeply stigmatized topic in many cultures, including Türkiye, which significantly hinders effective prevention efforts [3].

Stigma is defined as negative judgments towards individuals based on characteristics such as mental illness, ethnicity, or disability [4]. Individuals with psychological disorders often face frequent stigmatization and labeling [5]. This stigma extends to those exhibiting suicidal behavior, who are often perceived as cowardly, selfish, or weak-willed [6, 7]. Stigma surrounding suicide is a complex issue rooted in ignorance and misconceptions [8]. In many cultures, suicide is seen not just as a personal failing but also as a moral and social transgression, leading to harsh judgments and social ostracization [9]. Such societal views can deter individuals from seeking help, presenting a significant barrier to suicide prevention [10–12].

Research indicates that health professionals often hold negative attitudes towards suicide, shaped by societal norms and personal beliefs. These attitudes can negatively affect the quality of care provided to suicidal patients and may discourage them from seeking help [13]. Furthermore, medical students, as future healthcare providers, display varying attitudes toward suicide influenced by their education and personal experiences. While some students have accurate information about suicide and are willing to help those who attempt suicide, others hold more stigmatizing attitudes [14].

Medical education plays a pivotal role in shaping the attitudes of future healthcare professionals. Studies have shown that exposure to and education about mental health and suicide significantly influence students’ attitudes [15]. For instance, increased personal exposure to suicide is associated with higher suicide literacy and confidence in managing suicidal patients [16]. Integrating comprehensive suicide prevention training in medical curricula is crucial for fostering more supportive and informed attitudes among medical students.

Therefore, understanding medical students’ attitudes towards suicide is critical, as negative attitudes among healthcare providers can lead to suboptimal care for suicidal patients, perpetuating the cycle of stigma and hindering effective intervention [17].

Several studies have examined the attitudes of healthcare professionals toward suicidal behavior. For example, a study from Puerto Rico found that suicide literacy and emotional reactions significantly predicted suicide stigma among medical students [18]. Another study highlighted that stigma levels toward psychiatric patients varied among medical and nursing students, with more positive attitudes observed among female students and those from countries with higher suicide literacy [17].

Various educational strategies have been tested to improve medical students’ knowledge and attitudes about suicide and to teach them how to manage suicide interventions. Interventions such as film screenings, the use of clinical vignettes, and simulation-based training have been incorporated into suicide education curricula, with promising short-term outcomes [19, 20]. However, the long-term sustainability of these attitude changes remains uncertain. Research specifically focusing on medical students in Türkiye is limited. This study aims to fill this gap by exploring the attitudes of Turkish medical students towards suicide and assessing how their medical training influences these attitudes.

This study aims to (1) assess Turkish medical students’ suicide knowledge and stigma, (2) examine how this changes during training, and (3) identify curricular opportunities for improvement.

The results of this study may have significant implications for medical training in Türkiye, providing insights into how curricula can be improved to foster more supportive and informed attitudes towards suicide among future healthcare providers. Building on this and similar research, initiatives can be launched to incorporate comprehensive suicide prevention education into medical school programs.

## Methods

### Study Context

Medical training in Türkiye consists of a six-year curriculum. The first three years are devoted to theoretical and laboratory education. The fourth and fifth years involve clinical internships, while the sixth year serves as a hands-on internship year (often called ‘intern’ year) in which students work in hospitals for 12 months under supervision. In their fifth year, students attend a two-hour theoretical course on suicide and suicide intervention as part of the psychiatry block. In their final year, students undertake a one-month psychiatry internship, where they participate in diagnosing and treating psychiatric disorders as part of the hospital team.

### Data Collection Tools and Implementation

This cross-sectional study focused on first–, fourth–, and sixth-year students at Ondokuz Mayıs University’s Medical Faculty. It included all students from these classes without sampling. The study was designed to include first, fourth, and sixth-grade students representing preclinical, early clinical, and post-psychiatric exposure periods.

Data were collected using a survey in Google Forms. The survey consisted of 96 items divided into two sections: the first section gathered sociodemographic information, while the second included the Literacy of Suicide Scale and the Stigma of Suicide Scale.

*The Literacy of Suicide Scale (LOSS)* was used to assess participants’ knowledge of suicide. The scale was originally developed by Calear, Batterham, and Christensen, and its first major application and detailed description in the literature was in the study by Chan et al. [21]. This 27-item instrument was designed to comprehensively assess four key domains of suicide literacy as conceptualized by Jorm [22]: (a) recognizing signs and symptoms, (b) understanding risk factors, (c) identifying causes and triggers, and (d) knowing treatment and prevention options. To build a robust foundation for the scale, its developers incorporated 12 items from the well-established Revised Facts on Suicide Quiz (RFOS) by Hubbard and McIntosh [23], supplementing them with additional items to ensure comprehensive coverage of all four domains. Each item is rated on a 3-point Likert scale (“True,” “False,” or "Don't know"). Correct responses are scored one point, resulting in a total score range of 0 to 27, with higher scores indicating greater suicide literacy.

For the purposes of this study, we used the Turkish version of the LOSS, which was translated, and its validity and reliability were established by Öztürk and Akın [24]. Although the original developers later published a refined 26-item version of the LOSS [25], the Turkish validation was performed exclusively on the original 27-item structure [24]. Therefore, to ensure cultural and linguistic appropriateness and to rely on a psychometrically validated tool for our target population, the 27-item Turkish version was employed. In the present sample, the Kuder–Richardson 20 (KR-20) coefficient for the total LOSS score was 0.63.

The Stigma of Suicide Scale (SOSS) was developed by Batterham et al. [26] and evaluates stigma associated with suicide using descriptors for an individual who has died by suicide. The Turkish version of the scale, validated by Öztürk, Akın, and Durna [27], consists of 55 items rated on a 5-point Likert scale (“Strongly agree,” “Agree,” “Neutral,” “Disagree,” “Strongly disagree”). The scale includes three factors: stigma towards individuals who have died by suicide, the association of suicide with isolation/depression, and normalization/glorification of suicide. Each factor is scored separately, with higher scores on the stigma factor indicating greater stigmatization. In this study, the Cronbach’s alpha coefficient for the total SOSS items was 0.90.

Formal permission for the use of the Turkish versions of both the Literacy of Suicide Scale (LOSS) and the Stigma of Suicide Scale (SOSS) was obtained from the respective researchers who conducted the Turkish validation and reliability studies [24, 27].

Students’ views on religious beliefs and their families’ economic status were assessed using 9-point Likert scales. Religious belief was measured with the question, "How religious do you consider yourself?" (1: Not religious at all, 9: Very religious). Economic status was assessed with the question, "How would you rate your family's financial situation?" (1: Very poor, 9: Very rich). The responses were classified as low (1–3), average (4–6), and high (7–9).

### Participants

Before the main study, the questionnaire was pre-tested with 10 students to identify potential issues. Minor adjustments were made based on the feedback to improve clarity. The survey was administered from March to May 2024. Students were informed through the Ondokuz Mayıs University Medical Faculty Learning Resources Center website, Yahoo group, email addresses, and WhatsApp groups, and were invited to complete the survey. Each participant could complete the survey only once.

All participating sixth-year students had completed their psychiatry internships.

There was no student among the participants in the study who stated that they had attempted suicide before.

### Statistical Analysis

Data were analyzed using IBM SPSS version 21.0 software (SPSS Inc., Chicago, IL, USA). Descriptive statistics were presented as means ± standard errors and percentages. The student’s t-test was used for comparisons between two groups, and analysis of variance (ANOVA) was used for comparisons involving more than two groups. Pearson’s correlation test was applied to evaluate relationships between religiosity, family economic status, and scale scores.

## Results

### Sociodemographic Characteristics

At the time of the study, there were 341 first-year, 359 fourth-year, and 257 sixth-year students studying at the medical faculty. A total of 684 students participated in the study: 240 (70.4%) from the first year, 266 (74.1%) from the fourth, and 178 (69.3%) from the sixth.

59.3% of the students participating in the study were female. The mean age of the students was 21.77 ± 0.13 years, and they were predominantly from urban, middle-class backgrounds (Table 1). There were no participants among the students who stated that they had attempted suicide. However, 16.5% of participants reported having previously considered suicide at some point in their lives.

**Table 1:** Distributions of various sociodemographic characteristics

		**Number (%)**
Gender	Male	276 (40.4)
	Female	406 (59.3)
	Other(s)	2 (0.3)
The type of area where the individual spent his childhood and adolescence	Villages	42 (6.2)
	Towns	232 (33.9)
	Cities	410 (59.9)
Maternal education level	Elementary	210 (30.7)
	Middle	174 (25.4)
	University	300 (43.9)
Paternal education level	Elementary	110 (16.1)
	Middle	137 (20.0)
	University	437 (63.8)
Mother’s employment status	Working	242 (35.4)
	Not working	373 (54.5)
	Retired	65 (9.5)
	Deceased	4 (0.6)
Father’s employment status	Working	494 (72.2)
	Not working	17 (2.5)
	Retired	146 (21.3)
	Deceased	27 (4.0)
Family economic status	Low	21 (3.0)
	Average	394 (57.6)
	High	269 (39.4)
Religiosity	Low	187 (27.4)
	Average	386 (56.4)
	High	111 (16.2)
Total		**684 (100.0)**

### Knowledge Levels

The distribution of LOSS scores by class year is presented in Table 2. The average correct response rate was 63.59%. Fourth- and sixth-year students had significantly higher total knowledge scores than first-year students (*p* < 0.05). However, there were no significant differences in knowledge of causes/triggers or treatment/prevention (*p* > 0.05). Common misconceptions included statements such as "People who attempt suicide do not plan it in advance," "Most people who die by suicide are under 30," and "The risk of suicide is highest when a person begins to recover from depression."

**Table 2:** Distribution of scale scores by students’ academic years

	Total (*n* = 684) Mean ± SE	Class			F	p*
		I (*n* = 240) Mean ± SE	IV (*n* = 266) Mean ± SE	VI (*n* = 178) Mean ± SE		
*Literacy of Suicide Scale*						
Signs (point min/max = 0–6)	2.62 ± 0.05	2.46 ± 0.07 ^b^	2.78 ± 0.12^a^	2.89 ± 0.13^a^	5.50	** < 0.01**
Risk Factors (point min/max = 0–7)	4.44 ± 0.05	4.28 ± 0.06^b^	4.58 ± 0.13^a^	4.73 ± 0.11^a^	6.74	**0.001**
Causes/Triggers (point min/max = 0–10)	6.54 ± 0.09	6.42 ± 0.12	6.58 ± 0.22	6.79 ± 0.17	1.51	0.22
Treatment/Prevention (point min/max = 0–4)	3.55 ± 0.03	3.54 ± 0.04	3.54 ± 0.07	3.62 ± 0.07	0.45	0.63
TOTAL (point min/max = 0–27)	17.17 ± 0.13	16.73 ± 0.17^b^	17.58 ± 0.31^a^	17.96 ± 0.28^a^	7.93	** < 0.001**
Stigma of Suicide Scale						
Stigma (point min/max = 28–140)	67.95 ± 0.79	71.47 ± 0.93^b^	62.77 ± 1.94^a^	63.04 ± 1.77^a^	14.87	** < 0.001**
Isolation/Depression (point min/max = 16–80)	60.19 ± 0.42	60.89 ± 0.57	58.68 ± 0.92	59.55 ± 0.88	2.11	0.12
Glorification/Normalization (point min/max = 11–55)	26.52 ± 0.32	26.82 ± 0.41	25.81 ± 0.67	26.28 ± 0.74	0.724	0.48

^*^ANOVA

^a^^−^^b^Different superscript letters within a row indicate statistically significant differences based on post-hoc tests (*p* < 0.05)

### Stigma Attitudes

Scores on the SOSS are detailed in Table 2. First-year students had significantly higher stigma scores compared to fourth- and sixth-year students (*p* < 0.05), although there were no significant differences in isolation/depression or glorification/normalization scores (*p* > 0.05). Common stigmatizing descriptors included "unnatural," "selfish," "unjustifiable," "pathetic," "hurtful," and "cowardly."

### Correlations and Additional Findings

A significant negative correlation was found between knowledge levels and stigma scores (r = −0.25, *p* < 0.001). Female students had significantly lower stigma scores than male students (t = 4.47, *p* < 0.001), but there was no significant gender difference in knowledge scores (*p* > 0.05). No significant differences in knowledge or stigma scores were observed based on the type of area where students spent their childhood and adolescence (*p* > 0.05).

Significant negative correlations were also observed between family economic status, religiosity, and knowledge scores regarding causes/triggers and total knowledge (*p* < 0.05). Additionally, higher religiosity was positively correlated with stigma scores and negatively correlated with glorification/normalization scores (*p* < 0.05) (Table 3).

**Table 3:** Correlations between students’ scale scores and their economic status and religiosity

Scales	Subscales		Family economic status (point min/max = 1–10)	Religiosity (point min/max = 1–10)
			Mean ± SE 6.10 ± 0.61	Mean ± SE 5.07 ± 0.12
*Literacy of Suicide Scale*	Signs	r	0.01	−0.07
		p	0.92	0.11
	Risk Factors	r	−0.05	0.01
		p	0.25	0.98
	Causes/ Triggers	r	−0.07	−0.12
		p	0.12	**0.01**
	Treatment/ Prevention	r	−0.01	0.01
		p	0.93	0.70
	Total	r	−0.07	−0.10
		p	0.14	**0.04**
Stigma of Suicide Scale	Stigma	r	0.06	0.21
		p	0.20	** < 0.01**
	Isolation/ Depression	r	−0.12	0.04
		p	**0.01**	0.36
	Glorification/ Normalization	r	0.07	−0.14
		p	0.15	** < 0.01**

## Discussion

In this study, the correct knowledge rate regarding suicide was 61.9% in the first year, 65.1% in the fourth year, and 66.5% among the sixth-year students. Studies from different countries have investigated participants’ knowledge levels regarding suicide using the LOSS scale in the general population [10, 28–32]. The mean level of correct responses to LOSS scale questions in these studies ranged between 55 and 63% [10, 28–32]. A study of nurses in Türkiye reported a correct response rate of 41% [33]. Another study involving Australian medical students reported a mean correct response rate of 64.8% among postgraduate students, 75.5% among undergraduate students, and 62.9% in a general university survey [21]. The level of correct responses among medical students was higher than in the general population, but still below what is expected of future physicians.

In this study, medical students’ knowledge about suicide increased during the preclinical period (first to third years), while their levels of stigmatization decreased. Our findings reveal a critical gap in the current medical curriculum: the transition to clinical training and the psychiatry internship fail to further enhance suicide literacy or mitigate stigma. This suggests that unstructured clinical exposure is an insufficient educational tool for this complex issue. Although there was no formal training on suicide during the first three years of medical education, the observed increase in students’ knowledge is likely attributed to the overall intellectual development fostered by the curriculum. No significant difference in knowledge levels was observed between fourth- and sixth-year students, despite the additional clinical exposure and psychiatry internships completed by the latter.

Students undergo a one-month psychiatric internship during their internships, where they meet patients who have attempted suicide and participate in their treatment. However, this experience did not affect their knowledge level or stigma level. Researchers have suggested that education and direct contact with individuals experiencing mental health problems can reduce stigmatization and increase literacy [34]. The findings of this study showed that the clinical period and psychiatry internship made no contribution to the increase in students’ knowledge levels concerning suicide or to the decrease in their stigmatization levels. This stagnation likely stems from three key factors. The first is that the training provided during the clinical period may not be sufficient to allow students to acquire knowledge. The second is that medical education may provide a certain level of knowledge, but once that level has been reached, no further improvement is achieved, regardless of additional education, and students may be unable to escape the cultural constraints of their society. Finally, unstructured exposure may not be effective in obtaining accurate information and reducing stigma. In all three cases, medical faculty training needs to be revised.

Therefore, a longitudinal, spiral suicide prevention program is recommended. Such a program must integrate small group discussions and lived experience perspectives. This program should expose students to suicide-related issues not only during psychiatry clerkships but across all stages of medical training. It should include small group discussions and workshops, incorporate the perspectives of individuals with lived experience, and offer educational activities that explore values, culture, and belief systems. The educational strategies we propose, specifically small group discussions and the incorporation of lived experience perspectives, are supported by evidence as effective means to address complex attitudes like stigma. Small group settings provide a safe space for students to explore their personal beliefs and confront misconceptions, facilitating deeper cognitive and affective engagement than traditional lectures. More importantly, contact-based education, which facilitates direct interaction with individuals who have lived experience of mental illness, is ranked among the most effective strategies for reducing stigma in educational settings [34]. This approach allows students to see beyond the "patient" label, fostering empathy and undermining stereotypical beliefs by personalizing the experience of suicide. This methodology directly targets the attitudinal roots of stigma that unstructured clinical exposure alone may fail to modify, offering a plausible solution to the lack of progress observed between the fourth and sixth years.

Levels of stigmatization in this study decreased as knowledge concerning suicide increased. A study from Germany also reported that stigmatization decreased as knowledge levels rose [32]. Community-based studies from various countries have also reported a negative correlation between stigmatization and levels of knowledge concerning suicide [35]. Possessing sufficient and accurate information about suicide can help prevent negative attitudes. Our findings also confirm this.

The students in this study most frequently described individuals who had attempted suicide with terms such as “unnatural,” “selfish,” “unjustified/unacceptable,” “pitiable,” “offensive,” and “cowardly.” In a previous study from Türkiye, the first four most agreed-upon items on the stigma sub-dimension were “he is strange,” “he is angry/prone to violence,” “he is a failure,” and “he is a sinner” [36]. The medical students in the present study exhibited less stigmatization toward suicides than other university students in Türkiye [36]. In a study from Australia, more than 25% of the participants agreed that individuals who die by suicide are “weak,” “reckless,” or “selfish” [26]. Another study found that a quarter of the sample agreed with the statement that individuals who die by suicide can be described as “irresponsible” or “cowardly” [21]. A study of Australian medical students reported that they most frequently described people who commit suicide as “selfish,” “reckless,” and “hurtful” [29]. The low acceptance level among medical students of stigmatizing terms such as “shameful/disgraceful,” “stupid/immoral,” “unforgivable,” and “sinful” to describe suicides is promising. Nevertheless, the study results still indicate the presence of prejudices that need to be addressed.

Women recorded significantly lower stigmatization of suicide scores than men in this study. Students’ levels of stigmatization regarding suicide also increased with their religiosity. Studies have reported higher levels of suicide-related stigmatization in men [32, 37]. A study from four different Arab countries found an association between religiosity and stigmatization of suicide [38, 39]. Considering these studies together with the present research, men and more religious individuals appear to hold more stigmatizing attitudes toward suicide. In Islam, suicide is absolutely forbidden and is regarded as an unforgivable sin [39]. The relationship between increased religious faith and increased stigmatization may derive from the Islamic view of suicide and the patriarchal nature of such societies. These findings are important because they show how varying cultural values across countries can influence the medical education process. Therefore, medical education programs should incorporate topics such as religious/societal/cultural norms, their impact on health and healthcare delivery, and how these values intersect with scientific knowledge.

Medicine has long been regarded as one of the most rewarding professions. However, being a physician also involves several stress factors, some specific to the medical field and others shared with other high-stress professions requiring high skill levels. Medical students face unique stresses and difficulties that extend beyond the academic curriculum. This may be one reason why health problems such as depression and burnout syndrome are frequently observed among medical students [40]. In this study, 16.5% of the participants reported having previously considered committing suicide at some point in their lives. A prior study from Türkiye reported severe suicidal ideation in 9.2% of medical students, suicide planning in 9.5%, and attempted suicide in 1.1% [41]. Studies regarding the incidence of attempted suicide among medical students are limited. However, one meta-analysis found that the incidence of suicidal ideation in the previous year among medical students was 11.1% [42]. Another study reported that 14% of medical students experienced suicidal ideation in their final year [43]. The students in the present research exhibited higher rates of suicidal ideation compared to other studies. This increase may be attributable to the psychosocial impact of the COVID-19 pandemic, including prolonged lockdowns, the transition to online education, severe restrictions on social life, and heightened uncertainties regarding academic progress.

This study has several relative limitations that should be considered when interpreting the results. The lack of a randomized sample and reliance on self-reported data may have introduced biases. Being cross-sectional, the study makes it difficult to draw conclusions about causality. Finally, the study was conducted at a single medical school, which may limit the generalizability of the findings. Future research should involve a more diverse and representative sample from multiple institutions to validate these results.

This is the first study to assess the effect of medical training in Türkiye on knowledge levels and stigmatization concerning suicide. The findings showed that medical students possess a better level of knowledge regarding suicide than Turkish society in general. Medical students’ knowledge levels increase significantly from the first year of education to the fourth. In parallel, stigmatizing attitudes decrease significantly in fourth-year students compared to those in their first year. However, once students progress to clinical training, become interns, come into contact with patients who have attempted suicide, and become involved in treatment, no improvement is observed in either their knowledge or stigma levels compared to the preclinical period. Additionally, male and more religious students were found to exhibit higher levels of stigmatization. This study provides compelling evidence that a fundamental redesign of the medical curriculum in Türkiye is necessary. Integrating the proposed longitudinal, contact-based educational strategies is crucial for cultivating a generation of physicians equipped to effectively address suicide without stigma.

## Data Availability

The data that support the findings of this study are available on reasonable request from the corresponding author. The data are not publicly available due to privacy or ethical restrictions.
